# Cryocompression Therapy for Recovery from Eccentric Exercise-Induced Muscle Damage in Healthy Young Men

**DOI:** 10.3390/sports13090290

**Published:** 2025-08-27

**Authors:** Shi-Di Lin, Trevor C. Chen, Hung-Hao Wang

**Affiliations:** Department of Physical Education and Sport Sciences, National Taiwan Normal University, Taipei 116, Taiwan; 81330022a@gapps.ntnu.edu.tw (S.-D.L.); tcchen@ntnu.edu.tw (T.C.C.)

**Keywords:** force sense, position sense, limb swelling, recovery after exercise, exercise performance

## Abstract

Background: Cryocompression, an emerging therapy combining cryotherapy and compression therapy, has limited evidence regarding its effects on recovery from exercise-induced muscle damage. Methods: This study aimed to compare the effects of cryotherapy (CT), normothermic water compression (NWC), and cryocompression (CC) on muscle damage, proprioception, and performance following eccentric exercise. Forty healthy male participants performed 30 sets of 10 maximal isokinetic eccentric contractions (30°/s) of the quadriceps of the non-dominant leg. Muscle damage indicators [thigh circumference (CIR), muscle soreness measured by visual analog scale (VAS)], proprioception [position sense (PS), force sense (FS)], and performance parameters [range of motion (ROM), maximal voluntary isometric contraction (MVIC)] were assessed before and on days 1–5 following eccentric exercise. Two-way repeated-measures ANOVA with Tukey’s post hoc tests was used to evaluate group × time interactions. Results: Significant interactions were observed for CIR between the CT, NWC and CC groups compared to the control group, as well as for VAS scores between the CC and CON groups (*p* < 0.05). No other outcome measures showed significant interactions (*p* > 0.05). The control group showed a peak CIR increase of ~6.6 mm (day 3) versus 2.4 mm (CT), 3.6 mm (NWC), and 2.1 mm (CC). By day 5, the control group remained elevated at 5.2 mm, while CT returned to baseline by day 4. NWC and CC groups showed no significant changes on days 1–5. VAS scores in the CON group peaked at ~77 mm on day 2, not returning by day 5, whereas the CC group reached 48 mm and returned to baseline by day 3. Conclusion: Cryocompression reduced limb swelling and muscle soreness, as well as post-exercise-induced muscle damage, and NWC mitigated limb swelling, but none significantly affected proprioception or performance parameters.

## 1. Introduction

When the human body engages in physical activity, particularly unfamiliar eccentric exercises, it is often associated with muscle damage and delayed onset muscle soreness (DOMS). These conditions lead to a decline in athletic performance, including reductions in strength and range of motion (ROM), muscle swelling, and elevated creatine kinase (CK) activity [1,2,3,4]. Additionally, performance impairments, such as reduced countermovement jump (CMJ) height and 30 m timed hop performance, have been observed [5,6], along with impaired proprioception [7,8]. Although these symptoms typically resolve within 10–14 days, strategies to mitigate muscle damage and enhance recovery are critical for athletes and physically active individuals. For competitive athletes, rapid recovery is essential not only to maintain performance in congested training and competition schedules but also to minimize the risk of subsequent injury and optimize long-term adaptation [9]. Therefore, identifying effective recovery strategies is of both practical and clinical importance.

Recovery methods following exercise include cryotherapy, dynamic and static compression therapy, and cryocompression therapy—a combination of cooling and pressure application [10]. Cryocompression therapy involves wearing a specialized sleeve fitted to a limb, which is filled with circulating ice water to provide cooling and connected to a machine that applies intermittent, cyclic pressure via air compression. From a mechanistic perspective, cryocompression integrates the advantages of cryotherapy and compression therapy. By mimicking the muscle pump effect, it enhances venous return, increases blood flow, and improves tissue perfusion. Furthermore, the circulating ice water cools the tissue and modulates inflammatory mediators such as histamine and prostaglandins, thus alleviating symptoms of DOMS [11,12,13].

Based on previous clinical applications and related studies, cryocompression therapy has been shown to reduce postoperative pain, blood loss, swelling, and wound severity, as well as enhance local blood flow [14,15,16,17,18]. However, some researchers have reported conflicting results [19,20,21]. Their studies demonstrated that cryocompression in postoperative care showed no significant differences relative to control groups in terms of pain, swelling, range of motion, and postoperative blood loss [19,20,21]. Although most evidence derives from clinical settings, a few studies have also examined cryocompression in athletic recovery contexts, typically comparing it with passive rest or no intervention, yet the findings remain inconsistent [10,22,23,24].

Only three studies to date have investigated cryocompression therapy for post-exercise recovery [10,23,24]. For example, Dupont et al. [10] evaluated the effects of cryocompression administered to the lower limbs after resistance exercise. The results showed that, compared to the control group (rest), cryocompression significantly enhanced CMJ power, reduced muscle soreness, and lowered creatine kinase levels. Based on these findings, the authors concluded that cryocompression could more effectively promote lower limb muscle recovery after traditional high-load resistance training compared to no intervention. However, contrasting results have been reported by other studies. For example, Alexander et al. [23] administered cryocompression to participants’ hamstrings after fatigue induced by the Yo-Yo Intermittent Recovery Test Level 1 (YYIR1). Their findings showed significant reductions in skin temperature but no significant changes in average or peak eccentric hamstring strength when compared to the control (rest) group. Similarly, in another study [24], cryocompression was administered to participants’ thighs after daily training sessions held three days post-match. The results indicated significant reductions in CMJ height and skin temperature but no significant changes in adductor isometric peak strength or hamstring flexibility when compared to the control (passive recovery) group.

Despite inconsistent findings across three studies [10,23,24], evidence suggests that cryocompression post-exercise may alleviate pain, reduce CK activity [10], lower tissue temperature [10,23,24], and enhance CMJ performance [10]. However, no studies to date have investigated the effects of cryocompression on recovery from eccentric exercise-induced muscle damage and impaired proprioception.

The present study aims to investigate the effects of cryocompression on recovery from eccentric exercise-induced muscle damage and impaired proprioception, compared with cryotherapy, normothermic water compression and the control condition (rest). We hypothesized that, following eccentric exercise-induced muscle damage, the cryocompression group would exhibit faster recovery across all outcome measures compared to the control, cryotherapy, and normothermic water compression groups. No significant differences were expected between the cryotherapy and normothermic water compression groups, but both were expected to outperform the control group.

## 2. Materials and Methods

### 2.1. Participants and Study Design

The present study was approved by the Research Ethics Committee of National Taiwan Normal University in Taiwan. The study was conducted in conformity with the policy statement regarding the use of human subjects by the Declaration of Helsinki, and the protocol was approved by the Ethics Committee of 202306HM019 on 31 July 2023. The participants were healthy young adult males aged 18–35 years who were not professional athletes but engaged in recreational physical activity approximately 2–3 times per week (e.g., casual sports or fitness activities), with no history of cardiovascular diseases, asthma, diabetes, cold-related disease (e.g., Raynaud’s disease), or other major disease. They had not experienced musculoskeletal injuries in the past six months and were not taking any performance-enhancing drugs. Eccentric exercise can induce a repeated bout effect (RBE)—a protective adaptation of skeletal muscle that reduces susceptibility to subsequent exercise-induced damage for several weeks [25]. Thus, a between-subjects design was selected instead of a crossover design to avoid carryover effects, cumulative fatigue, and RBE, which are frequent concerns in EIMD research. An estimated sample size of least 10 participants were necessary for each group, with an alpha level of 0.05 and power (1-β) of 0.95 (G*Power 3.1.9.7, Heinrich-Heine-Universität Dusseldorf, Dusseldorf, Germany). The data collection of the present study was performed between August 2023 and May 2024 over 9 months. 40 participants were recruited and randomly assigned to four groups (*n* = 10 per group). Their (mean ± SD; range) age was 21.7 ± 2.4 yrs, height was 176.5 ± 6.0 cm, body mass was 69.2 ± 9.9 kg, and body mass index was 22.2 ± 3.5 kg/m^2^.

**Figure 1:**
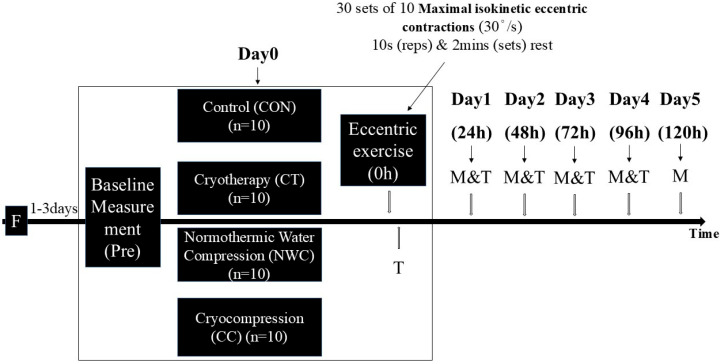
Study design (between-subjects with repeated measures). F: Familiarization, M: Measurement (Order as follows: CIR, ROM, Muscle soreness, Position Sense, MVIC (include RMS-EMG), Force Sense, CMJ height, 30 m Timed Hop), T: Treatment intervention (20 min).

Participants visited the laboratory on seven separate visits, including one familiarization session and six formal experimental sessions. A between-subjects design with repeated measures was used to evaluate the effects of cold therapy, normothermic water compression, and cryocompression on symptoms associated with eccentric exercise-induced muscle damage in the quadriceps of the non-dominant leg across days 1 to 5 post-exercise (Figure 1). The non-dominant leg was selected to minimize interference from habitual use. This limb-dominance definition is consistent with common practice in the literature, where dominance is often assigned based on preferred kicking or takeoff leg [26].

In a familiarization session that was set at one to three days before the official test, the participants were randomly assigned to four groups (10 participants per group), labeled as the control group (CON), cryotherapy group (CT), normothermic water compression group (NWC) and cryocompression group (CC). They experienced the measurements of upper thigh circumference (CIR), range of motion (ROM), muscle soreness, countermovement jump (CMJ) and 30 m timed hop test. MVIC of knee extensors, knee position sense, force sense and a single maximal isokinetic eccentric contraction (30°/s) were performed on an isokinetic dynamometer (Biodex System 4; Biodex Medical Systems, Shirley, NY). The collection of root mean square electromyography (RMS-EMG) data during MVIC was also conducted. However, to prevent the repeated bout effect [27] from occurring during eccentric exercise practice, which could provide a protective effect against the exercise intervention in subsequent formal experiments, the familiarization process for eccentric exercise involved only a demonstration by the examiner for the participants to observe.

At baseline (pre-exercise), participants completed the following sequence of tests: CIR, ROM, muscle soreness, knee position sense, MVIC, force sense, CMJ height and 30 m timed hop test. Root mean square electromyography (RMS-EMG) signals were collected during MVIC testing. After the baseline assessment, participants performed the eccentric exercise protocol, immediately followed by the 20 min treatment intervention according to their assigned group.

Post-exercise follow-up measurements were conducted at 24, 48, 72, 96, and 120 h after the eccentric exercise (days 1–5). At each follow-up, the same sequence of measurements as baseline was collected, followed by the corresponding 20 min treatment intervention.

To ensure consistency, all measurements and exercise sessions were supervised and administered by the same examiner. Throughout the study, participants were instructed to refrain from strenuous physical activity, maintain a consistent diet and hydration, and avoid other forms of treatment (e.g., supplements, medications, massage, icing).

### 2.2. Procedures

#### 2.2.1. Eccentric Exercise

Participants performed 30 sets of 10 maximal isokinetic eccentric contractions (30°/s) of the knee extensors of their non-dominant leg using a Biodex dynamometer (Biodex System 4; Biodex Medical Systems, Shirley, NY, USA) [28]. Previous research has demonstrated that this protocol effectively induces significant muscle damage [28]. The exercise was performed with participants seated on the Biodex chair at an angle of 85°, with the trunk, pelvis, and thigh securely strapped to minimize extraneous movements. The dynamometer arm was aligned with the lateral femoral epicondyle, and the lower leg was firmly attached with a padded cuff. The range of motion was set from 10° to 100° (0° representing full knee extension). Each contraction was initiated by the dynamometer moving the lever arm, while participants were verbally encouraged to maximally resist the imposed movement throughout the eccentric phase. A 10 s rest was given between repetitions, and a 2 min rest was provided between sets. A familiarization trial with several submaximal contractions was performed prior to the protocol to ensure correct execution.

#### 2.2.2. Treatment Intervention

The control group rested quietly in a supine position for 20 min. CT, NWC and CC groups received 20 min of treatment via an Aircast Cryo/Cuff IC (Aircast Cryo/Cuff IC Cooler; DJO Global, Vista, CA, USA) on their nondominant leg’s quadriceps 1, 25, 49, 73, and 97 h post-eccentric exercise. In the CT group, the pressure was set to 0 mmHg, and the temperature was maintained at 10 °C. In the NWC group, the pressure was set to 45–60 mmHg, and the temperature was maintained at 35 °C. In the CC group, the pressure was set to 45–60 mmHg, and the temperature was maintained at 10 °C. The parameters (duration, temperature and pressure) were determined based on the previous studies and device’s user manual [10,23,24,29].

#### 2.2.3. Muscle Damage Indicators

The measurement of Upper thigh circumference (CIR) was conducted by marking the center of the rectus femoris muscle belly with a permanent marker. Participants stood unilaterally on a low box, with the non-dominant leg relaxed and suspended in the air. A measuring tape was used to assess the marked location, ensuring that the tape remained horizontal and at the examiner’s eye level. Each assessment was conducted three times, with an allowable error margin of no more than 0.2 cm between measurements. If the error exceeded this threshold, the measurement was repeated. The final CIR value for this study was determined by calculating the mean of the three closest measurements [30].

The Range of motion (ROM) of the knee joint was assessed by measuring the relaxed (RANG) and flexed (FANG) knee joint angles using a 360° plastic goniometer. The protocol was adapted from a previous study [30]. The anatomical landmarks for the goniometer’s stationary arm, axis, and moving arm were the lateral midpoint of the femur, the lateral axis of the knee joint, and the lateral midpoint of the fibula, respectively. These landmarks were marked with a permanent marker before the eccentric exercise testing to maximize consistency throughout the study. Before measurement, participants stood on a low box, like the stance used for CIR measurement. The RANG was determined by instructing participants to relax their non-dominant leg, whereas the FANG was determined by instructing participants to maximally bend the knee to the greatest extent possible, aiming to bring the heel into contact with the glutes. The FANG and RANG were both measured three times, and an average was calculated for each angle. The ROM of the knee joint was calculated by subtracting the mean FANG from the mean RANG. Full extension is defined as 180 degrees.

Participants were assessed for muscle soreness using a Visual Analog Scale (VAS) at 24, 48, 72, 96, and 120 h (day 1–5) post-exercise. The VAS consisted of a 100 mm horizontal line, with the leftmost end (0 mm) representing “no pain at all” and the rightmost end (100 mm) indicating “extreme soreness” [31]. During the measurement, participants lay prone on a treatment table while the examiner supported the knee joint and ankle of the tested (non-dominant) leg with both hands. The examiner then performed one maximal passive extension and one maximal passive flexion of the knee joint to induce a sensation of soreness in the knee extensor muscles. Participants were then instructed to indicate their perceived muscle soreness level by marking a vertical line on the horizontal scale. The recorded distance in millimeters from the leftmost end of the scale was used as the measure of muscle soreness in this study [30].

Before the MVIC test, hair and skin were shaved with a razor, followed by cleansing with an alcohol wipe to remove oil and keratin until the skin exhibited mild hyperemia. Electrodes were then placed at the center of the vastus lateralis muscle belly of the participant’s non-dominant leg, with a 2 cm spacing between the positive and negative electrodes, while the ground electrode was placed above the ipsilateral patella [32].

The MVIC test was performed with participants seated on the Biodex, and they were encouraged to perform maximal isometric contractions (MVICs) of the non-dominant leg at a knee joint angle of 90°. Each participant completed three trials, each lasting 5 s, with a 45 s rest interval between trials. Verbal encouragement was provided throughout the test. The highest value recorded among the three trials was taken as the MVIC value [32]. The electromyographic (EMG) signal during MVIC was recorded, and the root mean square (RMS) at the peak torque (PT) was calculated [32]. In addition, neuromuscular efficiency (NME) was calculated by dividing the peak torque during MVIC by the corresponding RMS-EMG.

The Rate of force development (RFD) was calculated using MVIC of the knee ex-tensor muscles. The onset of muscle contraction was defined as the moment when torque exceeded 2.5% of the baseline torque up to peak torque (PT). RFD was determined as the slope of the torque–time curve (ΔTorque/ΔTime). RFD values were computed for 0–50 ms, 0–100 ms, and 0–200 ms intervals. Each participant performed three trials, and the highest recorded RFD value was used for analysis [33].

#### 2.2.4. Proprioception

Before proprioception testing, gravity correction was performed according to the operating manual. Then, the seat height, orientation, backrest tilt angle (85°), and neck support angle were adjusted according to the participant’s requirements. The knee joint ROM was set between 10° and 100° (with 0° indicating full knee extension). The stability of the securing straps used to fix the participant to the seat was rechecked before proceeding with the test.

The participant first underwent position sense testing. The limb (non-dominant leg) was then extended by the examiner at a slow steady speed (∼10°/s) from the 90° knee flexion position to 45° and held it for 10 s. This procedure was repeated three times, with a 30 s rest interval between trials, while instructing the participant to memorize the sensation of knee position at 45°. Afterward, the participant attempted to actively replicate the 45° knee position. When they perceived that their knee had reached the target angle, they pressed a handheld stop button to fix the current knee joint position. This was repeated five times, with a one-minute rest interval between trials [7]. The same procedure was then performed under a blindfolded condition to eliminate visual cues. The angle from the baseline (first test) was used as the initial angle, and the difference between the test result and the initial angle was calculated as the experimental outcome, with the average value employed for analysis.

Force sense was assessed using 30% of the participant’s baseline MVIC as the reference target. Firstly, they used the non-dominant leg to perform an isometric contraction while observing the screen displaying the reference line, aiming to match their contraction force to 30% of their baseline MVIC. Verbal guidance and encouragement were provided to help the participant maintain this force level. The test consisted of three trials, each lasting 10 s, with a one-minute rest interval between trials, allowing the participant to memorize their 30% MVIC sensation. Subsequently, the participant was blindfolded and instructed to replicate the 30% MVIC contraction intensity of their non-dominant leg without visual feedback. Five contraction trials were performed, with a one-minute rest interval between each. The contraction force of each trial was recorded, and the average value was calculated for analysis [7].

#### 2.2.5. Performance Tests

For the CMJ height, the smartphone was mounted on a tripod, and its position was adjusted to ensure the camera could fully capture the participant’s entire jump sequence. The participant stood upright with hands on hips. Upon hearing the tester’s command, they lifted their dominant leg and performed a single-leg CMJ using their non-dominant leg while the examiner recorded the entire jumping process. Each participant performed three jumps, with a 2 min rest interval between trials. My Jump 2.0 software (My Jump 2.0; My Jump Lab, Madrid, Spain) was used to calculate the participant’s jump height, and the highest recorded value was used for analysis [34].

For the 30 m single-leg timed hop, the protocol was adapted from a previous study [35]. The participant performed a single-leg hop using their non-dominant leg, covering a 30 m distance as quickly as possible. The built-in stopwatch function of a smartphone was used for timing. Each participant completed two trials, with a 2 min rest interval between trials. The best recorded time was used for analysis.

### 2.3. Statistical Analyses

Raw electromyographic (EMG) signal processing and analysis were performed using Acknowledge Version 3.8.1 software (Biopac Systems, Santa Barbara, CA, USA). A band-pass filter (20–500 Hz) was applied to the EMG signals obtained within 0.25 s before and after peak torque during each maximal voluntary isometric contraction (MVIC). The signals were then processed using full-wave rectification and smoothing, and the 0.5 s EMG segment was analyzed using the Root Mean Square (RMS) method. The RMS values corresponding to the MVIC peak from the three trials were used for further analysis.

For statistical analysis, the independent variables were grouped (CON, CT, NWC, CC) as the between-subject factor and time was grouped (pre-exercise, 24 h, 48 h, 72 h, 96 h, and 120 h post-exercise) as the within-subject factor. The dependent variables included:

Muscle damage markers: thigh circumference (CIR), muscle soreness, MVIC torque, RMS-EMG and NME values. Proprioception measures: knee position sense error and force sense error. Performance measures: countermovement jump (CMJ) height and 30 m hop test time.

To account for potential baseline variability, outcome measures such as MVIC, CMJ height, and force sense were expressed as delta values or percentage changes from baseline. This approach facilitates fairer between-group comparisons without requiring covariate adjustment. All statistical analyses were conducted using SPSS 23.0 for Windows. A two-way mixed-design analysis of variance (ANOVA) was employed to examine the effects of different interventions on changes in muscle damage indicators, proprioception, and performance parameters across different time points (pre-test, days 1–5 post-exercise). If a significant interaction effect (*p* < 0.05) was detected, Tukey’s post hoc test was conducted for pairwise comparisons. If no significant interaction was observed, a main-effect analysis was performed. The significance level was set at *p* ≤ 0.05. The data were presented as mean ± SEM.

## 3. Results

### 3.1. Baseline Measurements

All variables at the baseline (before the exercise) were not significantly (*p* > 0.05) different among four groups.

### 3.2. Muscle Damage Indicators

**Figure 2:**
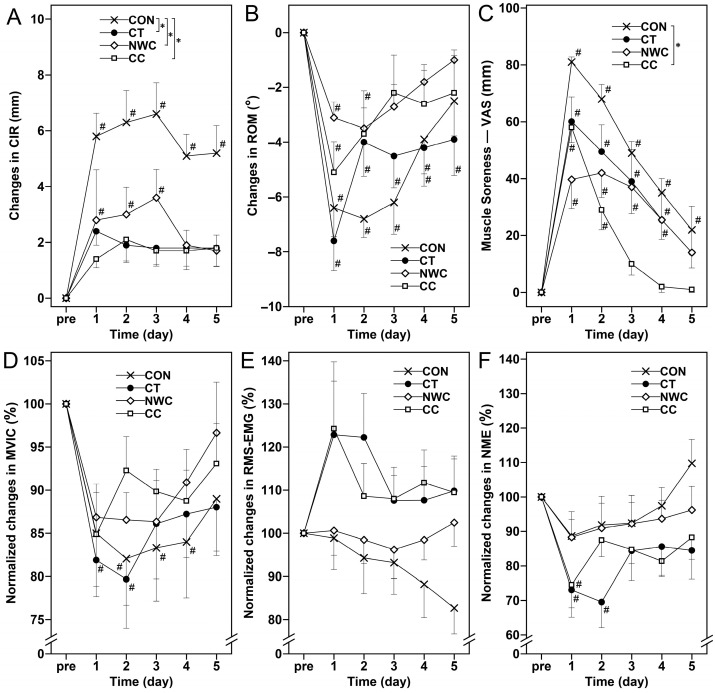
Changes (mean ± SEM) in upper thigh circumference (CIR, (**A**)), range of motion (ROM, (**B**)), muscle soreness assessed by a 100 mm visual analog scale (VAS, (**C**)), normalized maximal voluntary isometric contraction (MVIC, (**D**)), normalized root mean square electromyography (RMS-EMG, (**E**)), and normalized neuromuscular efficiency (NME, (**F**)) at baseline (i.e., pre), and 1, 2, 3, 4, and 5 days (1–5) after the eccentric exercise performed across four different groups. *: a significant (*p* < 0.05) interaction effect determined by two-way mixed ANOVA. #: a significant (*p* < 0.05) difference from baseline (i.e., pre) value.

A significant group × time interaction was observed for thigh circumference (CIR) (F = 24.438, η^2^ = 0.191, *p* < 0.05). Post hoc comparisons revealed that increases in CIR were significantly greater in the CON group compared with the CT, NWC, and CC groups (*p* < 0.05), with no significant differences among the three treatment groups (CT vs. NWC, CT vs. CC, NWC vs. CC; *p* > 0.05) (Figure 2A).

For muscle soreness, a significant interaction effect was also detected (F = 87.979, η^2^ = 0.198, *p* < 0.05). The CC group reported significantly lower soreness compared with the CON group (*p* < 0.05), whereas no significant differences were found between CT and CON, NWC and CON, or among the intervention groups themselves (*p* > 0.05) (Figure 2C).

**Figure 3:**
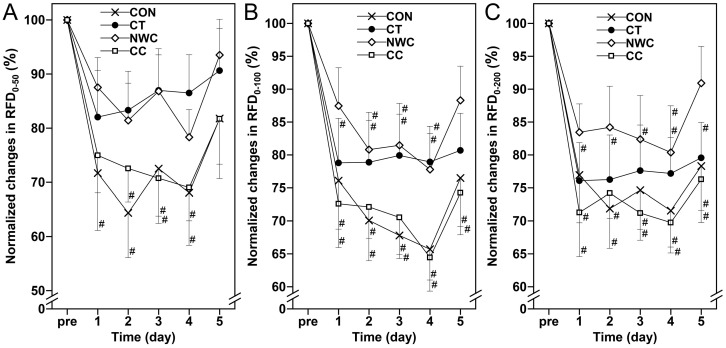
Changes (mean ± SEM) in normalized RFD_0–50_ (**A**), RFD_0–100_ (**B**) and RFD_0–200_ (**C**) at baseline (pre), and 1, 2, 3, 4, and 5 days (1–5) after the eccentric exercise performed across four different groups. #: a significant (*p* < 0.05) difference from baseline (i.e., pre) value.

Regarding time effects, CIR in the CON and NWC groups (Figure 2A), MVIC in the CON and CT groups (Figure 2D), RMS-EMG in the CON group (Figure 2E), NME in the CT group (Figure 2F), and RFD_0–50_ in the CON and CC groups (Figure 3A) all changed significantly over time (*p* < 0.05). In addition, ROM (Figure 2B), muscle soreness (Figure 2C), RFD_0–100_ (Figure 3B), and RFD_0–200_ (Figure 3C) showed significant time-dependent changes across all groups (*p* < 0.05), while no significant changes were observed in the remaining measures (*p* > 0.05).

### 3.3. Proprioception

**Figure 4:**
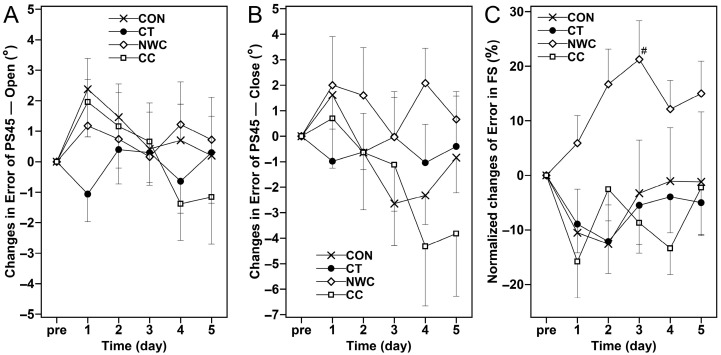
Changes (mean ± SEM) in error of knee position sense-open (PS45—Open, (**A**)), error of knee position sense-close (PS45—Close, (**B**)) and normalized error of force sense (FS, (**C**)) at baseline (pre), and 1, 2, 3, 4, and 5 days (1–5) after the eccentric exercise performed across four different groups. #: a significant (*p* < 0.05) difference from baseline (i.e., pre) value.

No significant time and interaction effect (*p* > 0.05) was observed for all measurements in proprioception (Figure 4).

### 3.4. Performance Parameters

**Figure 5:**
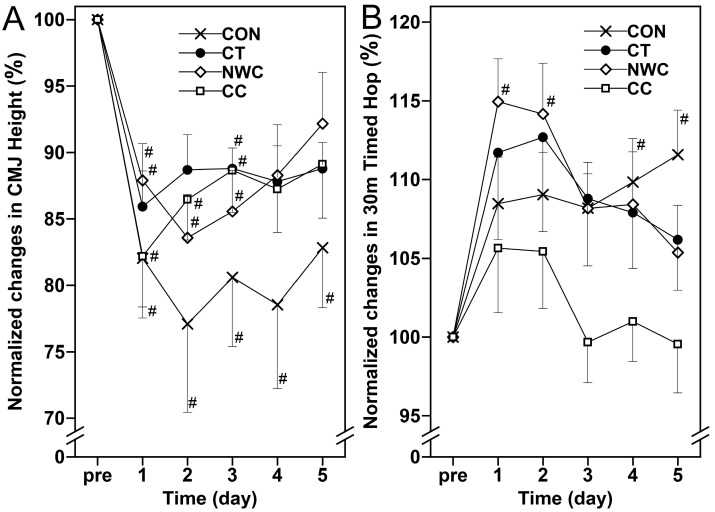
Changes (mean ± SEM) in normalized countermovement jump (CMJ) height (**A**) and 30 m timed hop test (**B**) at baseline (pre), and 1, 2, 3, 4, and 5 days (1–5) after the eccentric exercise performed across four different groups. #: a significant (*p* < 0.05) difference from baseline (i.e., pre) value.

No significant interaction effect (*p* > 0.05) was observed for all measurements in performance parameters (CMJ height and 30 m timed hop). CMJ height changed significantly over time for all groups (*p* < 0.05), with significant changes also observed over time in the 30 m timed hop for the CON and NWC groups (*p* < 0.05). However, no significant changes were found for the other conditions (*p* > 0.05) (Figure 5).

### 3.5. Summary Table

To enhance clarity, a summary table was created to highlight the main significant findings across groups and time points (Table 1). This table provides an overview of the key between-group and within-group differences observed for the primary outcome measures.

## 4. Discussion

We hypothesized that cryocompression therapy could enhance post-exercise recovery outcomes, such as reducing limb swelling and muscle soreness, and the results partially supported this hypothesis. Changes in CIR were significantly lower in the CT, NWC and CC groups compared to the CON group. Additionally, muscle soreness was significantly lower in the CC group relative to the CON group. However, no significant differences were observed among the groups in other measured outcomes.

### 4.1. Muscle Damage Indicators

To our knowledge, the present study is the first to administer cryocompression daily 24 to 120 h post-exercise-induced muscle damage and observe its effects on upper thigh circumference before and after the treatment intervention during those times. Our study demonstrated that using cryotherapy, normothermic water compression therapy, and cryocompression therapy all significantly reduced the increase in upper thigh circumference (Figure 2A). Maruyama et al. [36] applied a recovery intervention combining cold-water immersion and compression garments following eccentric exercise-induced muscle damage, finding a significantly lower upper thigh circumference in the experimental group compared to the control at 3 and 24 h post-exercise. This approach, which integrated cryotherapy and compression therapy to assess additive recovery effects, closely aligns with the mechanisms employed in our study and produced comparable outcomes.

An ongoing debate persists in the studies concerning the efficacy of cryotherapy alone, such as cold-water immersion (CWI) or partial-body cryotherapy (PBC), in reducing limb swelling following exercise-induced muscle damage. Hohenauer et al. [37] and de Freitas et al. [38] reported that neither PBC nor CWI significantly reduced upper thigh swelling following eccentric exercise-induced muscle damage. In contrast, Siqueira et al. [39] found that CWI significantly reduced upper thigh circumference after similar exercise protocols. In a related vein, studies investigating the effects of intermittent pneumatic compression (IPC) alone on swelling following muscle damage have produced inconsistent results. For example, Chleboun et al. [40] observed reduced upper arm swelling after eccentric exercise with IPC, whereas Waller et al. [41] reported no significant reduction in thigh or calf circumference following shuttle-run-induced muscle damage. These inconsistent findings indicate that the effectiveness of cryotherapy or IPC alone in reducing upper thigh circumference remains unclear, likely due to variations in exercise protocols (e.g., intensity, duration) and treatment parameters (e.g., temperature, compression levels) across studies. Future research should investigate the effects of cryocompression therapy on post-exercise limb swelling, considering standardized protocols and alternative measurement techniques, such as ultrasound, to enhance accuracy in assessing limb swelling.

In terms of ROM, our experimental results revealed no significant between-group differences. However, we observed a significant difference over time, with recovery rates ranked as follows, NWC group > CC group > control CON group > CT group (Figure 2B). In addition, this is the first study to investigate the effects of cryocompression on ROM after exercise-induced muscle damage. Previous research has reported mixed findings: some studies showed cryotherapy improved ROM [18,42,43], while others reported no effect of cold-water immersion or ice massage [44,45,46]. Similarly, IPC alone yielded inconsistent results, with no effect in some trials [47] but positive outcomes in others [48]. Taken together, the effects of cryotherapy or IPC on ROM remain inconclusive, and our findings highlight the need for further investigation of combined interventions such as cryocompression.

Cryotherapy and intermittent pneumatic compression (IPC) therapy have been shown to reduce limb swelling following exercise-induced muscle damage [41,49]. Given that limb swelling negatively correlates with joint range of motion (ROM) [40], cryocompression, which combines cryotherapy and compression, is theoretically anticipated to outperform standalone methods in reducing swelling and enhancing ROM. The hypothesis of the present study posited that cryocompression would yield superior reductions in limb swelling and improvements in ROM compared to other recovery methods, based on this theoretical framework. Contrary to expectations, the present study found that NWC therapy outperformed cryocompression in reducing limb swelling and improving ROM, challenging the initial hypothesis. Several factors may account for this discrepancy. First, cold likely influenced recovery outcomes; specifically, repeated cryocompression over five consecutive days post-exercise may have reduced tissue flexibility (e.g., muscles, tendons, ligaments) by inducing stiffness [11], thereby delaying ROM recovery. Second, individual physiological responses to temperature reductions in the cryotherapy and cryocompression groups likely varied due to factors such as cold sensitivity, resulting in greater inter-individual variability in ROM recovery compared to the NWC group.

The present study demonstrated that muscle soreness was significantly reduced following cryotherapy, NWC therapy, and cryocompression therapy (Figure 2C). On day 1 post-exercise, muscle soreness in the CT and NWC groups was significantly lower than in the CON group (CON: 77.0 ± 14.2 mm; CT: 52.5 ± 20.2 mm; NWC: 45.5 ± 20.3 mm). These findings are similar to previous studies [41,50]. Importantly, muscle soreness in the CC group was significantly lower than in the CON group on days 1, 2, 3, and 4 post-exercise (e.g., day 1: 48.0 ± 15.0 mm vs. 77.0 ± 14.2 mm), aligning with Dupont et al. [10], which observed sustained soreness reduction with cryocompression. In the present study, the maximum reduction in muscle soreness reached 39% in the CC group by day 4 post-exercise (from 49.0 ± 7.8 mm to 10.0 ± 7.8 mm), surpassing the 24% reduction reported by Dupont et al. [10]. Despite using the same visual analog scale (VAS), several biases may have influenced the results. First, muscle soreness, being a subjective measure, varies widely between individuals due to differences in pain perception. Second, the extent of muscle damage induced by the protocol may have varied due to factors such as individual strength or fatigue levels. However, the present study’s lack of objective muscle damage markers (e.g., creatine kinase) prevented quantification of muscle damage extent.

There were no significant differences in MVIC among the groups (Figure 2D). In the study of Alexander et al. [24], cryocompression was applied to the thighs of soccer players on the third day after a soccer match, following routine training. The results indicated no significant changes in the MVIC of the adductor muscles. This finding supports our experimental results, indicating that cryocompression has no significant effect on MVIC. Previous studies have indicated that neither cryotherapy nor IPC therapy effectively promotes power recovery following exercise [37,44,47,51]. These experimental findings support our results, from which it can be inferred that NWC therapy and cryocompression may have limited effects on power recovery following exercise-induced muscle damage. Currently, the effects of cryocompression on post-exercise strength recovery remain inconclusive due to insufficient experimental evidence. Additional experiments are required to substantiate its effects.

The results showed no significant between-group differences in RMS-EMG and NME (Figure 2E,F). This is the first study to examine the effects of cryocompression on these neuromuscular parameters following exercise-induced muscle damage. Previous studies on cryotherapy [52,53] and compression therapy [54] similarly reported no significant effects on EMG activity, while evidence on IPC remains lacking.

Although group differences in NME were not significant, recovery in the CT and CC groups was slower than in the CON and NWC groups, returning to baseline on days 3 and 2, respectively. Comparable findings have been reported in cold-water immersion studies, where NME recovery was delayed despite no significant between-group differences [55]. This may reflect reduced muscle force-generating capacity at low temperatures, requiring earlier recruitment of high-threshold motor units and leading to decreased NME [56]. Further research is warranted to clarify how cryocompression influences neural function during recovery.

The results showed no significant between-group differences in RFD. Previous studies on CWI have mostly reported no significant effects on isometric squat RFD [57,58], although some findings suggested faster RFD recovery in sport-specific jumps [59]. Similarly, studies comparing IPC and compression reported inconsistent outcomes despite similar protocols [60,61]. These discrepancies may stem from differences in testing methods and exercise models.

In our study, recovery patterns varied across groups: NWC demonstrated the fastest recovery (returning to baseline across all RFD indices by day 5), whereas cryotherapy showed delayed recovery and cryocompression did not appear to provide additional benefits. This suggests that mechanical compression may facilitate neuromuscular recovery without the inhibitory effects of low temperature, while the combination of cold and compression could potentially attenuate recovery.

Overall, the evidence indicates that the effects of cryotherapy and IPC on RFD remain inconclusive, highlighting the need for further research across different performance tests to clarify these mechanisms.

### 4.2. Proprioception

The results for position sense (PS45) showed no significant differences between groups, whether the eyes open or closed. No previous research has investigated the effect of cryocompression on position sense after exercise-induced muscle damage. Previous studies have reported conflicting findings regarding the effects of cryotherapy or compression therapy alone on joint position sense [62,63,64,65,66].

In the present study, neither skin nor muscle temperature was measured. Nevertheless, all participants exhibited the usual objective signs of tissue cooling such as intense skin redness. The lack of significant effects of cryotherapy, NWC therapy, and cryocompression on knee PS may be explained by several factors. Firstly, the cooling depth or duration may have been inadequate, with superficial cooling unlikely to influence deeper proprioceptive receptors, and the 35 °C water used in NWC therapy may not elicit temperature alterations in tissues such as skin or muscle [29]. Secondly, in contrast to previous studies [63,67] that employed acute interventions, the present study used a non-acute intervention recovery, which means during testing, the cooling-induced effects may have entirely dissipated.

For force sense (FS), no significant between-group differences were observed, although the NWC group showed a transient improvement on day 3 post-exercise before returning to baseline. This pattern contrasts with the other groups, which initially declined and then recovered. Previous studies have similarly reported that cryotherapy does not significantly affect position or force sense [68,69,70], likely because diminished cutaneous and muscle afferents are partially compensated by joint receptor inputs [69,71]. In addition, Fyfe et al. [72] found that cold water immersion led to a reduction in post-exercise anabolic responses after a single resistance exercise session and increased the baseline levels of post-training protein degradation markers. This could be another reason why the NWC group recovers faster. In summary, the faster recovery in the NWC group may be attributed to the absence of cold-related inhibitory effects and to the activation of proprioceptors through mechanical compression, which may facilitate neuromuscular recovery.

Overall, the findings suggest that neither cryotherapy nor cryocompression substantially affects proprioception, whereas NWC may offer some benefit for force sense recovery. Further studies are needed to clarify these mechanisms.

### 4.3. Performance Parameters

For CMJ height, no significant between-group differences were observed. The CON group failed to recover by day 5 post-exercise, whereas the CT, NWC, and CC groups returned to baseline by day 4, indicating faster recovery with these interventions compared to control.

Our finding contrasts with Alexander et al. [24], which reported a 13% reduction in CMJ height. The present study did not measure skin or muscle temperature, precluding assessment of internal muscle temperature changes induced by cryocompression. In contrast, Alexander et al. [24] documented an 18 °C decrease in skin surface temperature following cryocompression, suggesting that this substantial drop (which is greater than in comparable studies), may have elicited physiological alterations in deeper muscle structures, potentially influencing neuromuscular biomechanics and contributing to the observed reduction in jump height. The interventions in this study were non-acute, whereas Alexander et al. [24] assessed outcomes immediately post-intervention; the acute decrease in tissue temperature may lead to some short-term effects. This methodological difference likely underlies the divergent results.

No significant differences were observed between the groups in the 30 m timed hop. Additionally, the 30 m timed hop in the CON group did not return to baseline levels by day 5 after exercise, while there were no significant changes in the 30 m timed single-leg jump for the CT and CC groups post-exercise. The NWC group showed recovery to baseline levels by day 3 after exercise. These findings suggest that, in terms of promoting recovery of the 30 m timed hop following eccentric exercise, the recovery speed ranking for the four groups was as follows, CT and CC groups > NWC group > CON group.

We observed that the time required to complete the 30 m timed hop in the CON group was significantly greater on days 4 and 5 post-exercise compared to the baseline value. The previous study indicates that muscle edema and functional impairment typically peak 4–5 days post-exercise [73], which supports the results observed in our control group. Additionally, the NWC group demonstrated significantly increased completion times for the 30 m timed hop on days 1 and 2 following exercise compared to the baseline value. This trend was not evident in the other groups. Heat water compression therapy can mitigate excessive edema and reduce secondary inflammatory responses (e.g., TNF-α and ROS release), thereby advancing the timing of the inflammatory peak [74]. This finding supports the results observed in the NWC group. And cryotherapy more effectively suppresses the inflammatory process of muscle damage, resulting in no significant time effect observed in the CT and CC groups.

Clinically, normothermic water compression may be preferable for promoting recovery after eccentric exercise, particularly when rapid restoration of neuromuscular function is required. Cryotherapy and cryocompression could still be useful when inflammation control is prioritized.

### 4.4. Limitations

There are several limitations in the present study. Firstly, the participants of the study were healthy young adult males aged 18–35 years; thus, the results of the study may not reflect female, youth, and other populations. Although the sample size met the minimum requirement of power analysis (n = 10 per group), the relatively small number of participants may still limit the generalizability of our findings. Future studies with larger cohorts are needed to confirm these results. Secondly, daily physical activity levels of participants were not monitored, which may have contributed to variability in the recovery outcomes of the interventions. Thirdly, post-exercise measurements were not conducted immediately following eccentric exercise, and specific records of eccentric exercise intensity (e.g., force levels per repetition) were not documented. Fourthly, the control group consisted of passive rest, while some evidence suggests that light exercise may facilitate recovery after muscle damage. We selected passive rest as the control condition to represent the most common recovery strategy used in daily practice and to allow for a clear comparison with cryotherapy, compression, and cryocompression interventions. Lastly, we did not measure tissue temperatures, such as those of the skin or muscle, nor were blood-related biomarkers (e.g., CK, IL-6) assessed. Future studies should incorporate monitoring of skin and muscle temperatures, as well as measurements of blood biomarkers such as creatine kinase, to elucidate the effects of cryocompression therapy on recovery following eccentric exercise-induced muscle damage. Additionally, further investigation into the optimal frequency, duration, and temperature of cryocompression therapy is warranted to determine their impact on recovery outcomes. Comparing the recovery effects of cryotherapy versus other interventions (cryotherapy, NWC therapy) would also provide valuable insights.

## 5. Conclusions

In conclusion, the present study found that cryotherapy and cryocompression therapy can alleviate limb swelling and muscle soreness after exercise-induced muscle damage, but they did not have significant effects on proprioception and performance parameters. Furthermore, it was observed that cryotherapy was not beneficial for the recovery of joint range of motion after exercise-induced muscle damage. NWC therapy alleviated limb swelling, but it may be detrimental to the accuracy of strength sense, potentially increasing the risk of injury.

## Figures and Tables

**Table 1:** Summary of main significant findings across groups and variables.

Variable	Main Finding (Interaction)	Main Finding (Time Effect/Interaction)	Recovery to Baseline
CIR	CT, NWC, CC < CON	↑ post-exercise in CON, NWC groups	CC group recovered fastest
ROM	Not significant	↓ post-exercise in all groups	Recovery rank: NWC > CC > CON > CT
Muscle soreness (VAS)	CC < CON	↑ post-exercise in all groups	CC showed greatest and most sustained reduction
MVIC	Not significant	↓ in CON and CT groups	CON—Day 5 CT—Day 3
RMS-EMG	Not significant	No significant changes	–
NME	Not significant	↓ in CT and CC groups	CT—Day 3 CC—Day 2
RFD	Not significant	↓ in all groups	NWC recovered fastest; CC slowest
PS (position sense)	Not significant	No significant changes	–
FS (force sense)	Not significant	NWC ↑ on Day 3	–
CMJ height	Not significant	↓ in all groups	CT/NWC/CC recovered by Day 4; CON not by Day 5
30 m timed hop	Not significant	↑ in CON on Day 4–5 ↑ in NWC on Day 1–2	NWC by Day 3; CON not by Day 5

Note: ↑: increase; ↓: decrease.

## Data Availability

The original contributions presented in the study are included in the article, further inquiries can be directed to the corresponding author.

## Abbreviations

The following abbreviations are used in this manuscript:

DOMS	Delayed Onset Muscle Soreness
ROM	Range of Motion
CK	Creatine Kinase
CMJ	Countermovement Jump
YYIR1	Yo-Yo Intermittent Recovery Test Level 1
RBE	Repeated Bout Effect
EIMD	Exercise-induced Muscle Damage
MVIC	Maximal Voluntary Isometric Contraction
NME	Neuromuscular Efficiency
RMS-EMG	Root Mean Square Electromyography
CIR	Circumference
RANG	Relaxed Angle
FANG	Flexed Angle
VAS	Visual Analog Scale
PT	Peak Torque
RFD	Rate of Force Development
ANOVA	Analysis of Variance
CWI	Cold-Water Immersion
PBC	Partial-Body Cryotherapy
IPC	Intermittent Pneumatic Compression
PS	Position Sense
FS	Force Sense
